# Secondary Intracranial Tumors Following Radiotherapy for Pituitary Adenomas: A Systematic Review

**DOI:** 10.3390/cancers9080103

**Published:** 2017-08-08

**Authors:** Ryuya Yamanaka, Eisuke Abe, Toshiteru Sato, Azusa Hayano, Yasuo Takashima

**Affiliations:** 1 Laboratory of Molecular Target Therapy for Cancer, Graduate School for Medical Science, Kyoto Prefectural University of Medicine, Kyoto 602-8566, Japan; ahayano@koto.kpu-m.ac.jp (A.H.); ytakashi@koto.kpu-m.ac.jp (Y.T.); 2 Division of Radiation Oncology, Niigata University Graduate School of Medical and Dental Sciences, Niigata 951-8122, Japan; eabe@med.niigata-u.ac.jp; 3 Department of Radiology, Nagaoka Chuo General Hospital, Nagaoka 940-8653, Japan; tsato515@nifty.com

**Keywords:** glioma, meningioma, pituitary adenoma, radiation-induced intracranial tumor, sarcoma

## Abstract

Pituitary adenomas are often treated with radiotherapy for the management of tumor progression or recurrence. Despite the improvement in cure rates, patients treated by radiotherapy are at risk of development of secondary malignancies. We conducted a comprehensive literature review of the secondary intracranial tumors that occurred following radiotherapy to pituitary adenomas to obtain clinicopathological characteristics. The analysis included 48 neuroepithelial tumors, 37 meningiomas, and 52 sarcomas which were published between 1959–2017, although data is missing regarding overall survival and type of irradiation in a significant proportion of the reports. The average onset age for the pituitary adenoma was 37.2 ± 14.4 years and the average latency period before the diagnosis of the secondary tumor was 15.2 ± 8.7 years. Radiotherapy was administered in pituitary adenomas at an average dose of 52.0 ± 19.5 Gy. The distribution of pituitary adenomas according to their function was prolactinoma in 10 (7.2%) cases, acromegaly in 37 (27.0%) cases, Cushing disease in 4 (2.9%) cases, PRL+GH in 1 (0.7%) case, non-functioning adenoma in 57 (41.6%) cases. Irradiation technique delivered was lateral opposing field in 23 (16.7%) cases, 3 or 4 field technique in 27 (19.6%) cases, rotation technique in 10 (7.2%) cases, radio surgery in 6 (4.3%) cases. Most of the glioma or sarcoma had been generated after lateral opposing field or 3/4 field technique. Fibrosarcomas were predominant before 1979 (*p* < 0.0001). The median overall survival time for all neuroepithelial tumors was 11 months (95% confidence intervals (CI), 3–14). Patients with gliomas treated with radiotherapy exhibited a non-significant positive trend with longer overall survival. The median overall survival time for sarcoma cases was 6 months (95% CI, 1.5–9). The median survival time in patients with radiation and/or chemotherapy for sarcomas exhibited a non-significant positive trend with longer overall survival. In patients treated with radiotherapy for pituitary adenomas, the risk of secondary tumor incidence warrants a longer follow up period. Moreover, radiation and/or chemotherapy should be considered in cases of secondary glioma or sarcoma following radiotherapy to the pituitary adenomas.

## 1. Introduction

Pituitary adenomas which can either be hormone secreting or non-secreting are benign neoplasms of the pituitary gland approximately account for 8% of all primary brain tumors. In pituitary adenoma patients (non-functioning adenomas or growth hormone (GH) and adrenocorticotropic hormone (ACTH) secreting adenomas), surgical management is the mainstay treatment. Radiotherapy is an option only if repeated surgery or medical treatment fails in patients with pituitary adenomas. Despite the improvement in cure rates, pituitary tumor patients treated with radiotherapy are at risk of long-term neurological complications such as the development of hypothalamic-pituitary dysfunction, leukoencephalopathy, and secondary malignancies [1,2,3,4,5,6]. Radiation-induced intracranial tumors might occur within the brain, the dura mater, leptomeninges, optic sheath, vascular adventitia, or the choroid plexus within the central nervous system (CNS), although it is rare. Tumors such as meningiomas, sarcomas, and gliomas are the most frequently reported secondary neoplasms [7,8,9,10,11]. Cahan et al. [12] established the diagnostic criteria for radiation-induced tumors. These criteria were modified by Schrantz and Araoz [13] as follows: (1) the tumor must occur within the irradiated field; (2) the tumor must not be present prior to the radiotherapy; (3) a sufficient latency period must elapse between irradiation and tumor incidence; and (4) the radiation-induced tumor must be proven to be of a different histological type compared to the original neoplasm treated by radiotherapy. We have reported the results of a systematic review of radiation-induced gliomas, meningiomas, and sarcomas in 728 patients [9,10,11]. Of these, 104 (14.3%) patients were derived from pituitary irradiation, and this is one of the major etiologies of radiation-induced brain tumor. In the present study, we focused on secondary intracranial tumors following radiotherapy to pituitary adenomas to obtain an insight into clinicopathological characteristics.

This review is based on a systemic search in the PubMed databases. The terms used in the search were “radiation induced intracranial tumor” combined with any of the following words: “brain tumor,” “glioma,” “glioblastoma,” “malignant glioma,” “anaplastic astrocytoma,” “meningioma,” “sarcoma,” “secondary neoplasm,” “pituitary adenoma,” “radiotherapy-induced,” “radiation-induced.” Cases with more than 3 years of latency period from radiation therapy to the secondary intracranial tumor diagnosis were selected. Several factors were collected, including patient age at diagnosis, gender, latency period from radiation therapy to the secondary brain tumor diagnosis, total irradiation dosage, irradiation method, pituitary adenoma type, location of secondary tumor, therapy of secondary tumor and overall survival (OS). OS was calculated from the date of secondary brain tumor diagnosis to the date of death, regardless of the cause of last follow-up. A Kaplan-Meier analysis was used to illustrate the OS. Statistical significance was assessed using a log-rank test. Hazard ratios and 95% confidence intervals (CI) from a logistic regression model were used to compare groups with respect to major clinical factors. A value of *p* < 0.05 was considered to indicate statistical significance.

## 2. Results

### 2.1. The Incidence of Intracranial Tumors Following Radiotherapy to Pituitary Adenomas Reported in the Literature

**Figure 1:**
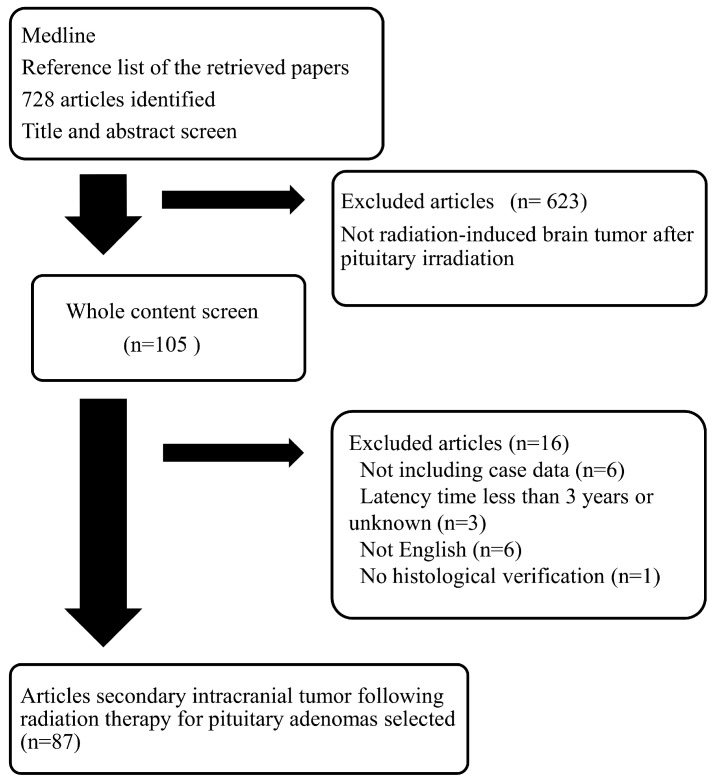
Flowchart of the selection process for studies included in the meta-analysis.

**Figure 2:**
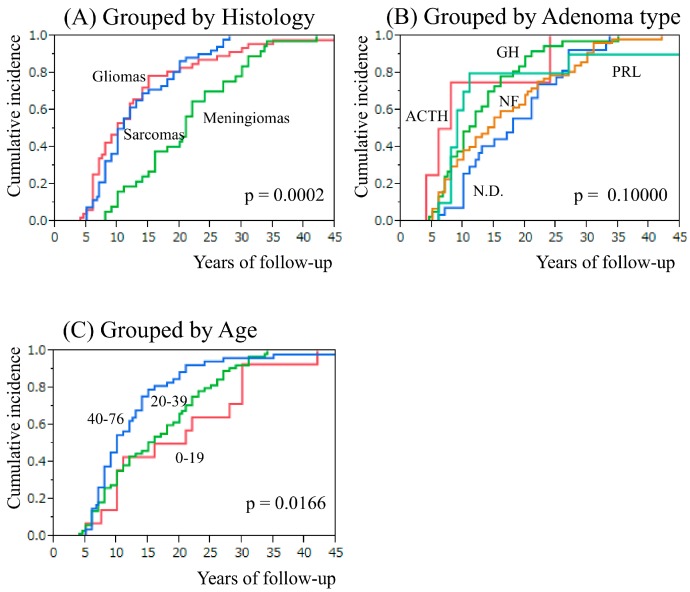
Latency period from pituitary adenoma to development of secondary brain tumor. (**A**) Comparing groups classified as brain tumor histology. (**B**) Comparing groups classified as pituitary adenoma type. ACTH: Cushing disease, GH: acromegaly, N.D.: not determined, NF: non-functioning adenoma, PRL: prolactinoma. (**C**) Comparing groups classified in 0–19, 20–39, 40–76 year age group.

**Figure 3:**
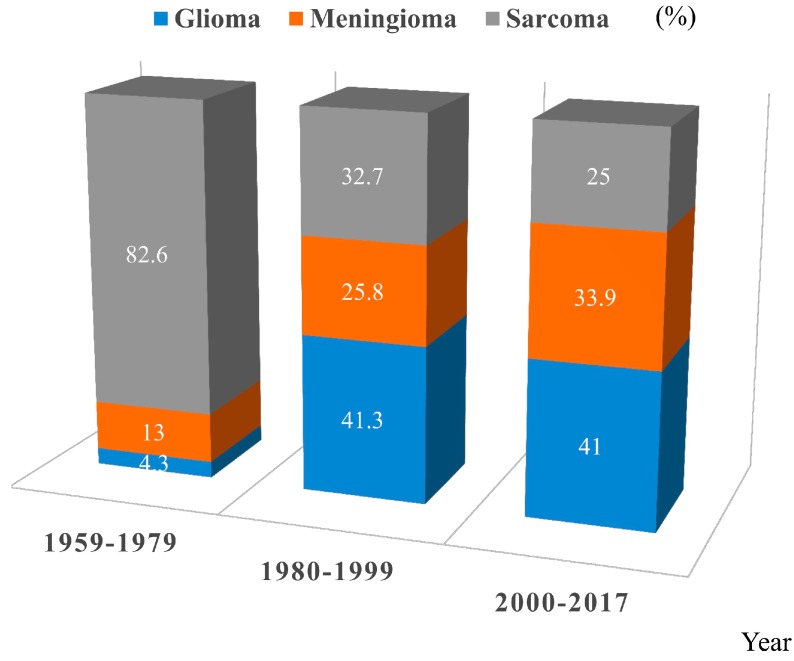
Three categories of second brain tumors were divided in year group, 1959–1979, 1980–1999, 2000–2017.

We initially identified 728 articles. After articles were excluded based on our present inclusion and exclusion criteria, finally 87 articles with a total of 137 (48 neuroepithelial tumors, 37 meningiomas, 52 sarcomas) patients in the 1959–2017 period were included in this review, although data is missing regarding overall survival and type of irradiation in a significant proportion of the reports (Figure 1, Table 1, Supplementary Table S1) [14,15,16,17,18,19,20,21,22,23,24,25,26,27,28,29,30,31,32,33,34,35,36,37,38,39,40,41,42,43,44,45,46,47,48,49,50,51,52,53,54,55,56,57,58,59,60,61,62,63,64,65,66,67,68,69,70,71,72,73,74,75,76,77,78,79,80,81,82,83,84,85,86,87,88,89,90,91,92,93,94,95,96,97,98,99,100,101,102]. The distribution of the histological types of secondary tumors was gliomas in 46 cases (9, grade II; 14, grade III; and 22, grade IV), primitive neuroepithelial tumor (PNET) in 1 case, neuroblastoma in 1 case, meningiomas in 37 cases (6, meningothelial; 5, fibroblastic; 1, transitional; 1, atypical; 1, malignant; 23, meningioma not specified), sarcomas in 52 cases (28, fibrosarcoma; 9, osteosarcoma; 3, malignant histiocytic sarcoma; 2, leiomyosarcoma; 1, fibrochondrosarcoma; 1, hemangioendothelioma; 1, malignant peripheral nerve sheath tumor; 8, sarcoma not specified) (Supplementary Table S1). The age of radiotherapy administered for pituitary adenomas was 39.7 ± 13.4 years for neuroepithelial tumors, 31.3 ± 13.4 years for meningiomas, and 39.0 ± 15.0 years for sarcomas (neuroepithelial tumors vs. meningiomas, *p* = 0.0042; meningiomas vs. sarcomas, *p* = 0.0130; Table 1). The latency period from radiotherapy administration to the onset of secondary tumors was 13.3 ± 9.1 years for neuroepithelial tumors, 20.9 ± 8.5 years for meningiomas, and 12.8 ± 6.5 years for sarcomas (neuroepithelial tumors vs. meningiomas, *p* < 0.0001; meningiomas vs. sarcomas, *p* < 0.0001; Table 1, Figure 2A). The radiation dose delivered to the pituitary adenomas was 51.3 ± 10.9 Gy for neuroepithelial tumors, 50.9 ± 23.7 Gy for meningiomas, and 53.2 ± 22.0 Gy for sarcomas (not significant; Table 1). Fibrosarcomas were predominant before 1979 (*p* < 0.0001; Figure 3). There were two cases of multiple secondary brain tumors with different histological types: meningioma and glioma with an 8-year interval [95] and fibrosarcoma and osteogenic sarcoma with a 4-year interval [77].

### 2.2. Secondary Intracranial Tumor and Adenoma Type or Age at Pituitary Irradiation

The distribution of pituitary adenomas according to their function was prolactinoma (PRL) in 10 (7.2%) cases, acromegaly (GH) in 37 (27.0%) cases, Cushing disease (ACTH) in 4 (2.9%) cases, thyrotropinoma (TSH) in 1 (0.7%) case, non-functioning adenoma (NF) in 57 (41.6%) cases, PRL + GH in 1 (0.7%) case and not determined (ND) in 27 (19.7%) cases. Secondary tumor developed in PRL, ACTH, GH, NF pituitary adenomas 9, 7, 11, and 14 years after irradiation, respectively (GH vs. NF, *p* = 0.0386; ACTH vs. NF, *p* = 0.1786; PRL vs. NF, *p* = 0.8318) (Figure 2B). Secondary tumor developed in 0–19, 20–39, 40–76 year age group was 18.5, 15, and 10 years after irradiation, respectively (*p* = 0.0166) (Figure 2C).

### 2.3. Irradiation Method and Secondary Tumor Location

**Figure 4:**
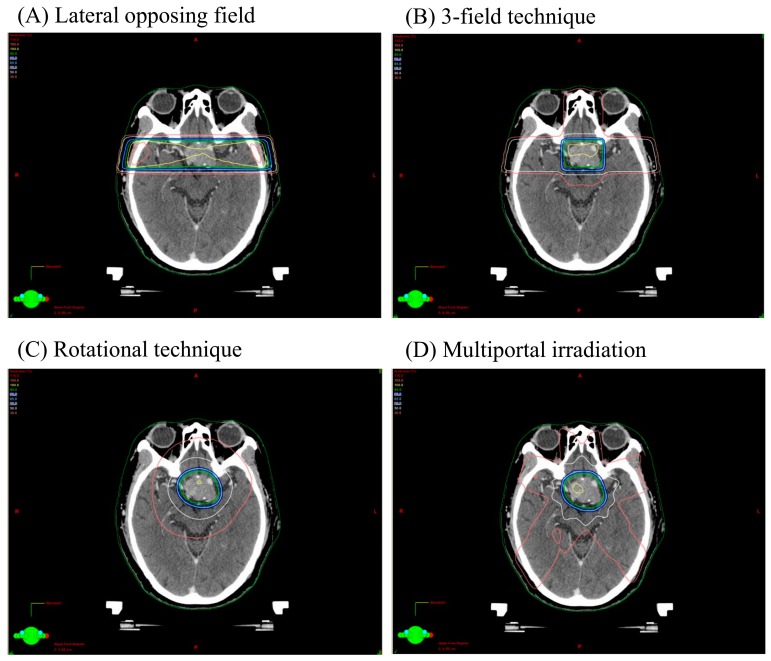
Typical isodose plan of lateral opposing field (**A**), 3-field technique (**B**), rotational technique (**C**), multiportal irradiation (**D**) for a case of non-functioning pituitary adenoma. The lesion dose is 4500 cGy/25 fractions and isodose lines corresponding to 30%, 50%, 80%, 85%, 90%, 95%, 100% of the target dose are shown.

Irradiation technique delivered was lateral opposing field in 23 (16.7%) cases, 3 or 4 field technique in 27 (19.6%) cases, rotation technique in 10 (7.2%) cases, radiosurgery in 6 (4.3%) cases and, N.D. (not determined) in 71 (51.8%) cases (Table 2). The location of the secondary tumor was frontal lobe in 14 (10.2%) cases, temporal lobe in 17 (12.4%) cases, parietal lobe in 3 (2.1%) cases, basal ganglia in 2 (1.4%) cases, chiasma in 3 (2.1%) cases, brainstem in 3 (2.1%) cases, skull base in 64 (46.7%) cases, calvaria in 15 (10.9%) cases and, N.D. in 24 (17.5%) cases. A typical isodose plan of lateral opposing field, 3-field technique, rotational technique, multiportal technique used for a case of non-functioning pituitary adenoma is shown in Figure 4. The lesion dose is 4500 cGy/25 fractions. The relationship of radiation technique and secondary brain tumor development is shown in Table 2. Most of glioma or sarcoma had occurred after lateral opposing field or 3/4 field technique.

### 2.4. The Result of Secondary Neuroepithelial Tumor Therapy

**Figure 5:**
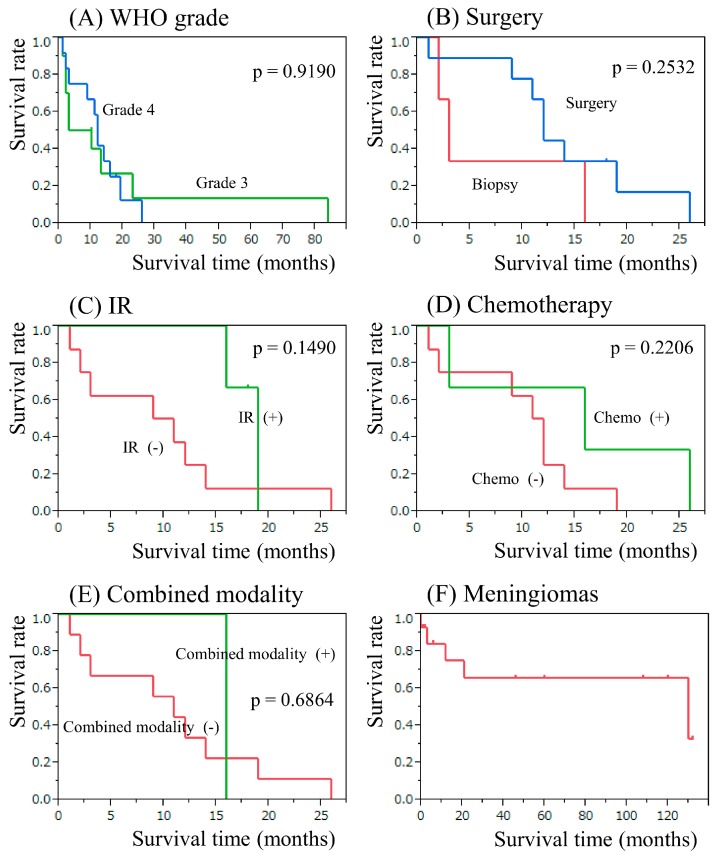
Kaplan-Meier survival analysis in patients with secondary gliomas. (**A**) Grouped by WHO grade. (**B**) Comparing groups classified by total or subtotal removal [Surgery] or biopsy [Biopy]. (**C**) Comparing groups classified by with irradiation [IR (+)] or without irradiation [IR (−)]. (**D**) Comparing groups classified by with chemotherapy [Chemo (+)] or without chemotherapy [Chemo (−)]. (**E**) Comparing groups classified by with combined modality [Combined modality (+)] or without combined modality [Combined modality (−)]. (**F**) Kaplan-Meier survival analysis in patients with secondary meningiomas.

In the case of secondary neuroepithelial tumor, total or partial removal was performed in 20 cases, and biopsy was performed in 7 cases. Radiotherapy was performed in 6 cases with an average dose of 53.1 (30–76) Gy. Chemotherapy was prescribed for 6 cases with a range of protocols according to the physician’s choice. The median overall survival time and 2-year survival rate for grade III and IV gliomas were 6.5 months (95% CI, 1.2–23) and 13.3%, 12 months (95% CI, 2–19), and 12.5%, respectively (Figure 5A). The survival time was also estimated depending on the treatment modality administered to grade IV gliomas. The median survival time in patients who underwent total or subtotal surgical removal was 12 months (95% CI, 1–19) with a 2-year survival rate of 16.6% and that in patients who underwent biopsy only was 3 months (95% CI, 2–16) with a 2-year survival rate of 0% (*p* = 0.2532, Figure 5B). The median survival time following radiotherapy was 19 months (95% CI, 16–19) with a 2-year survival rate of 0%, whereas patients who did not receive radiation had a median survival time of 10 months (95% CI, 1–14), and a 2-year survival rate of 12.5% (*p* = 0.1490, Figure 5C). The median survival time in patients who received chemotherapy was 16 months (95% CI, 3–26) with a 2-year survival rate of 33.3%, while patients who did not receive chemotherapy had a median survival time of 11.5 months (95% CI, 1–14) and a 2-year survival rate of 0% (*p* = 0.2206, Figure 5D). In patients who received surgery and radiochemotherapy, the median survival time was 16 months (95% CI, not reached (NR)) with a 2-year survival rate of 0%, whereas that for the remainder of the patients who had not received combined treatment was 11 months (95% CI, 1–19) with a 2-year survival rate of 11.1%, respectively (*p* = 0.6864, Figure 5E).

### 2.5. The Results of Secondary Meningioma and Sarcoma Therapy

**Figure 6:**
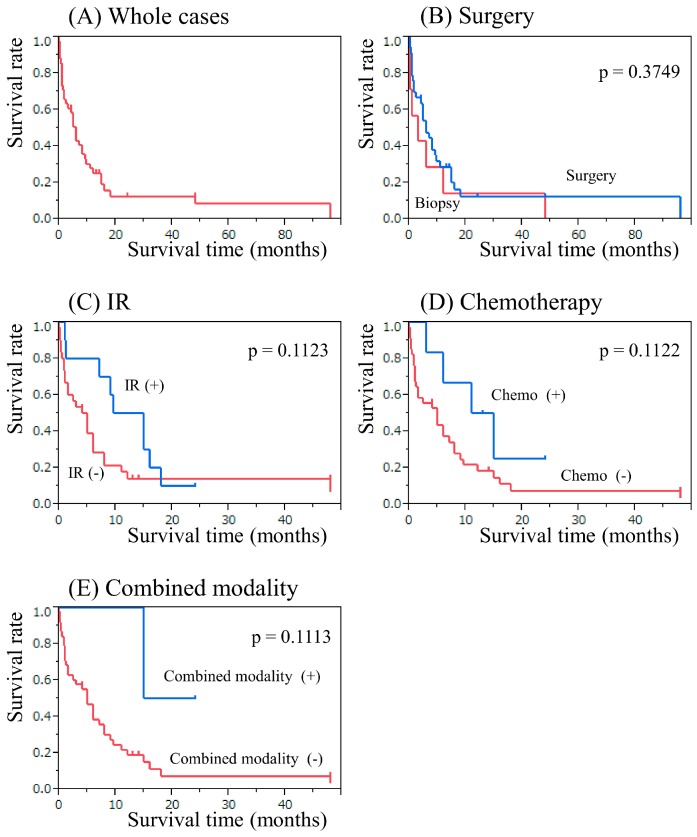
Kaplan-Meier survival analysis in patients with secondary sarcomas. (**A**) Whole cases. (**B**) Comparing groups classified by total/subtotal or partial removal [Surgery] or biopsy [Biopy]. (**C**) Comparing groups classified by with irradiation [IR (+)] or without irradiation [IR (−)]. (**D**) Comparing groups classified by with chemotherapy [Chemo (+)] or without chemotherapy [Chemo (−)]. (**E**) Comparing groups classified by with combined modality [Combined modality (+)] or without combined modality [Combined modality (−)].

In case of secondary meningioma, surgical resection was performed in 21 cases. The median overall survival time for all meningioma cases was 130 months (95% CI, 12–NR) with a 10-year survival rate of 65.6% (Figure 5F). There are various treatment options for secondary sarcoma, including surgery, chemotherapy, and radiotherapy. Total or partial resection of the tumors was performed in 36 cases, and biopsy was performed in 7 cases. Radiotherapy was administered for 10 cases with an average radiation dose of 46.1 (28–65) Gy. Chemotherapy was administered in 6 cases with a range of protocols according to the physician’s choice. The median overall survival time for all sarcoma cases was 6 months (95% CI, 1.5–9) with a 2-year survival rate of 12.7% (Figure 6A). The median survival time in patients who underwent total or subtotal surgical resection was 6 months (95% CI, 2.5–9.5) with a 1-year survival rate of 28.6%; and that in patients who underwent biopsy only was 3 months (95% CI, 0.06–12) with a 1-year survival rate of 14.2% (*p* = 0.3749; Figure 6B). There was no impact of surgery on secondary sarcoma. The median survival time in patients who received radiotherapy was 12.2 months (95% CI, 1–16) with a 1-year survival rate of 50.0%, whereas that in patients who did not receive radiotherapy was 4.5 months (95% CI, 1–6) with a 1-year survival rate of 14.2% (*p* = 0.1123, Figure 6C). The median survival time in patients who underwent chemotherapy was 13 months (95% CI, 3–NR) with a 1-year survival rate of 50%, while patients who did not receive chemotherapy had a median survival time of 5 months (95% CI, 1–8) and a 1-year survival rate of 18.6% (*p* = 0.1122, Figure 6D). In patients who underwent combination of chemotherapy and radiotherapy, the median survival time was NR months (95% CI, 15–NR) with a 1-year survival rate of 100%; whereas that for the patients who had not undergone radiochemotherapy was 5 months (95% CI, 1.5–8) with a 1-year survival rate of 19.3% (*p* = 0.1113, Figure 6E).

## 3. Discussion

In this series, all cases fulfil the criteria of radiation induced tumor. They occurred within the irradiated field; a sufficient latency period elapsed between irradiation and secondary tumor occurrence (at least 3 years) [103]; the secondary tumors were proven to be of a different histological type from the original neoplasm. Pituitary irradiation is one of the important treatment options for functioning and non-functioning adenomas. Radiotherapy is administered in the case of incomplete resection, invasive tumors, or tumors showing resistance to treatments. The use of radiotherapy is well established and effective in delaying tumor regrowth. Using X-ray simulation, radiotherapeutic techniques such as lateral opposing technique, 3-field technique, rotation technique, and radiosurgery had been employed individually to localize uniform radiation to the sellar region and minimize dose to the optic pathways, brainstem, and temporal lobes. The most important dose-limiting factor is the effect on optic pathways and brain. Sheline et al. had reported that the optimal dose range for maximal effects on primary tumor management, with minimal probability of side effects, was 40–50 Gy delivered in fractions <2 Gy per day [104,105]. Sellar region irradiation had been performed to deliver an approximate dose of 45 Gy in fractions. Radiation-induced gliomas or meningiomas had occurred often in the temporal and frontal region. In this series, approximately 75% of gliomas arose in the fronto-temporal region. Pituitary irradiation was often administered via bilateral opposed ports with or without the anterior beam, thereby exposing the temporal lobes to relatively high doses of radiation although this had been the older technique but not with the more contemporary treatment modalities. The incidence of secondary glioma might be enhanced by radiological exposure for the subependymal layer where neural stem cells are present after the lateral opposing field or 3-field technique. The older radiotherapy techniques/higher doses per fraction, favored the development of sarcomas, since most of the sarcomas occurred in patients treated between 1959–1979. Loeffler et al. reported meningioma occurrence after radiosurgery for pituitary adenoma in the region that received a very low estimated dose (4.4 Gy) of radiation [81]. We should consider secondary tumors, since they can occur when neighboring tissue receives even lower doses.

There are conflicting results as to the development of secondary intracranial tumors after pituitary irradiation [84,100,102]. Burman et al. reported the risk ratio for radiotherapy vs. no radiotherapy was 3.34 for malignant brain tumors, and 4.06 for meningiomas [102]. A connection between functional pituitary adenomas and the incidence of cancers has to be investigated, since certain hormones might act as promoters of oncogenesis following irradiation. Actually, patients with acromegaly have been shown to have increased incidence of malignant tumor [106,107,108], as shown in our data the latency period is shorter in patients with GH secreting adenoma compared to that of non-functioning adenomas. The association between functioning pituitary adenoma and secondary intracranial tumor is unclear. Growth hormone and insulin-like growth factors (IGFs) have been reported to be linked to the risk of cancers [109,110]. IGFs promote the proliferation of oligodendrocytes, and are believed to have a role in brain tumor progression [111]. IGF2 gene is overexpressed in gliomas and meningiomas [112]. Several insulin-like growth factor-binding proteins (IGFBPs), which regulate the bioactivity of IGFs are also expressed in brain tumor. Enhanced gene expression of IGFBP1 and IGFBP2 has been demonstrated in meningiomas and gliomas [113]. IGFBP2 is associated with increased cellular proliferation, migration, and invasion in glioblastomas [114,115]. Sex steroids regulate meningioma progression through IGFs and IGFBPs [116]. A mild hyperprolactinaemia is found in some of the patients with meningiomas [117]. PRL receptors are expressed in meningioma, astrocytic tumors, and osteoblastic cells, PRL being implicated as a regulator of proliferative effect of menigiomas, gliomas, and sarcomas [117,118,119]. There are also clinical findings of accelerated growth of co-existing meningiomas in patients with PRL secreting pituitary adenomas [120] and also accelerated growth of meningiomas during pregnancy [121]. Cushing disease results from excessive secretion of glucocorticoid, and patients are immunosuppressed due to hypercorticolism. Intracranial tumor development might be enhanced by the immunosuppressive effect. Although hormone production might have contributed to the brain tumor development, the precise hormonal follow-ups following radiotherapy for pituitary adenomas were not described in these reports.

The latency period from radiation exposure to the occurrence of secondary meningioma was longer compared to that for glioma or sarcoma, possibly owing to the slowly growing nature of the tumor. Spontaneous high-grade gliomas typically affect adults and are preferentially located in the cerebral hemispheres. However, the age of onset of radiation-induced gliomas is lower than that of spontaneous high grade gliomas [9]. In radiation-induced gliomas after pituitary irradiation, onset age is rather older (53 year) compared to radiation-induced gliomas in general (27 year), owing to the fact that the age of pituitary irradiation is rather older. Radiation-induced gliomas are frequently located in the cerebellum and spinal cord, whereas in radiation-induced gliomas after pituitary irradiation, frontal or temporal sites are predominant, probably due to the radiation method.

Radiation-induced meningiomas have been reported to be associated with a lower patient age at diagnosis, a higher number of calvarial tumors, histologically atypical/multiple lesions, and aggressive biological behavior in the course of the disease [10]. In radiation-induced meningioma after pituitary irradiation, onset age is also rather older (53 year) compared to radiation-induced meningioma in general (36 year), owing to the fact that the age of pituitary irradiation is rather older. A lower number of calvarial tumors after pituitary irradiation (21.6%) were identified compared to radiation-induced meningiomas in gereral (51%) probably due to the radiation method. The incidence of grade II and III meningiomas (13.3%) is lower compared to that in patients with radiation-induced meningiomas in general (31.7%), possibly due to lack of precise information.

The reports concerning the genetic alterations in radiation-induced intracranial tumors are limited. Briefly, somatic mutations of *p53* gene have been identified in radiation-induced tumors [122,123]. An initial mutation to a single allele might occur after radiotherapy, and an additional mutation to the wild-type allele might be added after several years, leading to oncogenesis. Nine radiation-induced high-grade gliomas were investigated for molecular alterations in *p53, PTEN, KRAS, EGFR*, and *p16* [74]. Genetic alterations similar to those described in spontaneous high-grade gliomas, with the exception of *PTEN* mutations, were also observed in radiation-induced gliomas. Loss of chromosome 1p was frequent in radiation-induced osteosarcomas and meningiomas [124,125,126]. However, a more wide-scale analysis, using a larger series, is required to truly address these issues.

Radiation-induced glioma is difficult to treat; radiotherapy is not always a therapeutic option because it could be that the patient already had prior exposure. However, Mayer and Sminia reported that re-irradiated normal brain tissue could tolerate a cumulative total dose of more than 100 Gy at conventional fractionation [127]. These observations indicate that higher doses are possible with re-irradiation. Therapeutic approaches by radiation might therefore yield prolonged disease control in some patients with radiation-induced tumor. Patients with neuroepithelial tumors treated with radiotherapy exhibited a non-significant positive trend with longer overall survival. Retrospective data supports the need for combined modality treatment in patients with secondary gliomas as reported [9]. The most effective treatment for radiation-induced meningiomas is surgical management, although radiosurgery could also be considered. Although the median survival time in patients with radiotherapy and/or chemotherapy for sarcomas had a tendency to be improved, treatment of radiation-induced sarcomas is much more difficult [11]. Surgical management had limited roles including decompressing mass effect, obtaining target space for radiotherapy, and sampling for pathological diagnosis, because most of the tumors had been adherent and invading to the surrounding structures and could only be partially removed. In our previous study, it was proposed that patients with radiation-induced sarcoma may benefit from intensive chemotherapy [11]. Berkmann et al. reported the usefulness of radiosurgery to obtain significant tumor shrinkage for a limited time [89]. Radiosurgery applies a high dose of radiation to a delineated space with a small risk of causing injury to the neighboring structures [128]. Alexandru et al. reported a case with relatively long-term disease control with cyclophosphamide and imatinib with no limiting toxicities [88]. With recent advances of molecularly targeted therapy for cancer, new agents are emerging for sarcomas.

Consistent and long-term aftercare is basically required in the case of pituitary adenomas not only to detect a possible radiation-induced tumor, but also to check the tumor control and to detect long-term damage to the optic pathway and the pituitary function. The follow-up should be performed in specialized centers with available radiation therapists, neurosurgeons, endocrinologists, and ophthalmologists.

There are limitations to this study, since the data were obtained from retrospective case reports and case series. However, our data supports clinicopathological characteristics of secondary intracranial tumors after irradiation for pituitary adenoma. The high proportion of sarcomas in this review may reflect a publication bias of the most severe tumors. This cannot only be due to different radiation protocols, this might be a problem of histological diagnosis. After irradiation therapy for pituitary adenomas, the risk of radiation-induced tumors will be increased in the longer survivors. Future studies should focus on genetic profiling of radiation-induced tumors to elucidate features that might aid in the development of targeted therapies.

## 4. Conclusions

The risk of secondary intracranial tumors should be considered before the decision of radiotherapy for treating pituitary adenomas. In addition, we suggest the importance of long-term follow up in patients who undergo radiotherapy for pituitary adenomas. Moreover, combined modality therapy should be considered in cases of secondary sarcoma following radiotherapy to the pituitary adenomas. Extensive molecular pathological research on radiation-induced tumors is warranted.

## Figures and Tables

**Table 1:** Characteristics of secondary tumor, Average (95% CI).

Secondary Tumor	Number of Cases	Age at Irradiation	Irradiation Dose (Gy)	Latency (Years)
Neuroepithelial Tumor	48	39.7 ± 13.4 (35.7–43.6)	51.3 ± 10.9 (47.5–55.2)	13.3 ± 9.1 (10.6–15.9)
Meningioma	37	31.3 ± 13.4 (26.7–36.0)	50.9 ± 23.7 (41.7–60.1)	20.9 ± 8.5 (18.1–23.8)
Sarcoma	52	39.0 ± 15.0 (34.7–43.3)	53.2 ± 22.0 (46.4–59.9)	12.8 ± 6.5 (11.0–14.6)
Total	137	37.2 ± 14.4 (34.7–39.7)	52.0 ± 19.5 (48.2–55.8)	15.2 ± 8.7 (13.7–16.6)

**Table 2:** Radiation technique and secondary brain tumor, n (%).

Radiation Technique	Neuroepithelial Tumor	Meningioma	Sarcoma	Total
Lateral opposing field	10 (20.8)	4 (10.8)	9 (17.3)	23 (16.7)
3 or 4 field technique	9 (18.7)	5 (13.5)	13 (25.0)	27 (19.6)
Rotational technique	2 (4.1)	6 (16.2)	2 (3.8)	10 (7.2)
SRS	1 (2.0)	2 (5.4)	3 (5.7)	6 (4.3)
N.D.	26 (54.1)	20 (54.0)	25 (48.0)	71 (51.8)
Total	48	37	52	137

N.D.: not determined; SRS: stereotactic radiosurgery.

## Supplementary Materials

The following are available online at http://www.mdpi.com/2072-6694/9/8/103/s1. Table S1: Radiation-induced secondary intracranial tumor following radiotherapy for pituitary adenomas.
